# Southern right whale *Eubalaena australis* in Golfo San Matías (Patagonia, Argentina): Evidence of recolonisation

**DOI:** 10.1371/journal.pone.0207524

**Published:** 2018-12-19

**Authors:** Magdalena Arias, Mariano A. Coscarella, M. Alejandra Romero, Nicolás Sueyro, Guillermo M. Svendsen, Enrique A. Crespo, Raúl A. C. González

**Affiliations:** 1 Centro de Investigación Aplicada y Transferencia Tecnológica en Recursos Marinos Almirante Storni (CIMAS,CONICET), San Antonio Oeste, Río Negro, Argentina; 2 Centro de Estudios de Sistemas Marinos (CESIMAR, CENPAT, CONICET), Puerto Madryn, Chubut, Argentina; 3 Universidad Nacional de la Patagonia San Juan Bosco (UNPSJB), Puerto Madryn, Chubut, Argentina; 4 Escuela Superior de Ciencias Marinas (ESCIMAR, UNCO), San Antonio Oeste, Río Negro, Argentina; Duke University, UNITED STATES

## Abstract

Since the 1980s, the distribution range of the southern right whale (*Eubalaena australis*) in Argentina was mostly located in the winter calving grounds around Península Valdés. After the international moratorium that forbade the commercial hunting, southern right whales have shown signs of recovery during the last few decades. Nowadays, it is thought that the species is experiencing a density-dependent process while expanding its distribution range in Patagonia. From 2007 to 2016, data on right whale distribution, group composition and relative abundance were collected in Golfo San Matías, Patagonia through aerial surveys. Generalized linear models with a negative binomial error distribution were used to determine the population trend of right whales in this area. In addition, the group composition and the relative abundance of right whales among the northern Patagonian gulfs were compared. Finally, a literature review was conducted to assess the historical presence of right whales in Golfo San Matías, revealing the presence of right whales in Golfo San Matías during and after the commercial exploitation. During aerial surveys (2007–2016), right whales were observed from August to October in the area, with a peak in late August-early September. Our results suggested a geographic distribution change with a regular use of the northwest coast of the gulf in recent years and a positive trend in the population growth rate inside Golfo San Matías. This area was dominated by unaccompanied whales (solitary individuals and breeding groups) as opposed to Península Valdés where the dominant group type was the mother calf pairs. Therefore, Golfo San Matías appears to be important for socializing and mating but not as a nursery ground. In addition, the density of whales was four times greater in the gulfs of Península Valdés. Our findings contribute to a better understanding of the recovery of this species in Patagonia, Argentina and should be considered for the management measures for right whales in this region.

## Introduction

During the eighteenth and nineteenth centuries commercial whaling caused a drastic decrease in the number of southern right whales (SRW) *Eubalaena australis* bringing them to the brink of extinction [1] with only 200–300 individuals left by 1920 [1,2]. The species has been internationally protected since 1935 by the International Convention for the Regulation of Whaling. However, between 1951 and 1972, the Soviets carried out illegal pelagic whaling, and particularly in the Southwest Atlantic these catches occurred in front of the coasts of Patagonia, Argentina [3], slowing the increase in whale numbers. Since the 1960s and 1970s, several stocks of whales off Argentina-Brazil, South Africa, New Zealand and Australia have shown signs of recovery [4–9].

In the Southwest Atlantic Ocean, there are two well-known SRW wintering grounds: Península Valdés (42–43°S), which has two major gulfs (Golfo Nuevo and Golfo San José) [10], and Santa Catarina (between Florianópolis Island, 27°25’S, and Laguna, 28°36’S) in Southern Brazil [11]. There is evidence of SRW movement between these two areas [12]. Although female SRW show fidelity to their calving grounds [10, 13] the species shows plasticity in its behaviour [12] and is able to expand its range and recolonise areas such as was reported in New Zealand [8] and South Africa [14]. Therefore, areas with suitable environmental conditions around these wintering grounds could be colonised by the species. This hypothesis is supported by more frequent sightings of SRW in recent years in Rio Grande do Sul in Brazil [9], Uruguay [15, 16] and Golfo San Matías (GSM) in Argentina [17–19]. In the latter, the presence of whales has become so frequent that whale watching has taken place since 2012 [18].

In Argentina, long-term SRW studies were carried out around Península Valdés, where the species has been monitored since 1999 by coastal aerial surveys [20]. As a result of these data, Crespo *et al*. 2018 [20] reported a decline in the SRW population growth rate in Península Valdés during the last 10 years and a change in the proportion of the different group types observed in the nearshore area (between the coastline and 1500 meters from it). During the first years of sampling all the group types were found close to the shore. However, as the population grew the mother calf pairs remanied near the coastline (between the coastline and 500 meters from it), while the others group types were displaced to deep waters or to other less dense regions such as GSM, north of Península Valdés [20–22]. These changes were suggested to be related to density-dependent processes, and probably have changed the balance on the relative abundance of SRW in the nearshore area of Península Valdés [20, 22], as it was proposed by Payne in 1986 [10]. Therefore, it is important to assess if most of the whales are found in the nearshore area in GSM, in order to validate if in this new area the coastal aerial survey (the method commonly used to census right whales [20, 23]) is a good method to monitor the relative abundance of the species in the area.

It was proposed that in Argentina the species could be recolonising suitable environments that have been occupied prior to the exploitation process [20]. Given that in Argentina most research efforts have focused on the known nursery areas around Península Valdés, the distribution, abundance and seasonal occurrence of SRW in the GSM is practically undescribed. In this context, the objectives of this study were: 1) to collect historical records of the presence of SRW in the GSM, 2) to validate the coastal aerial surveys as a good indicator of relative abundance of whales in the GSM, 3) to analyze the relative abundance and distribution of the species in the GSM, 4) to estimate the growth rate in this area, 5) to compare the proportion of group types and the relative abundance of whales among the northern Patagonian gulfs (GSM and Península Valdés system) to infer the process that goes through the GSM on a regional scale.

## Materials and methods

### Study area

**Fig 1:**
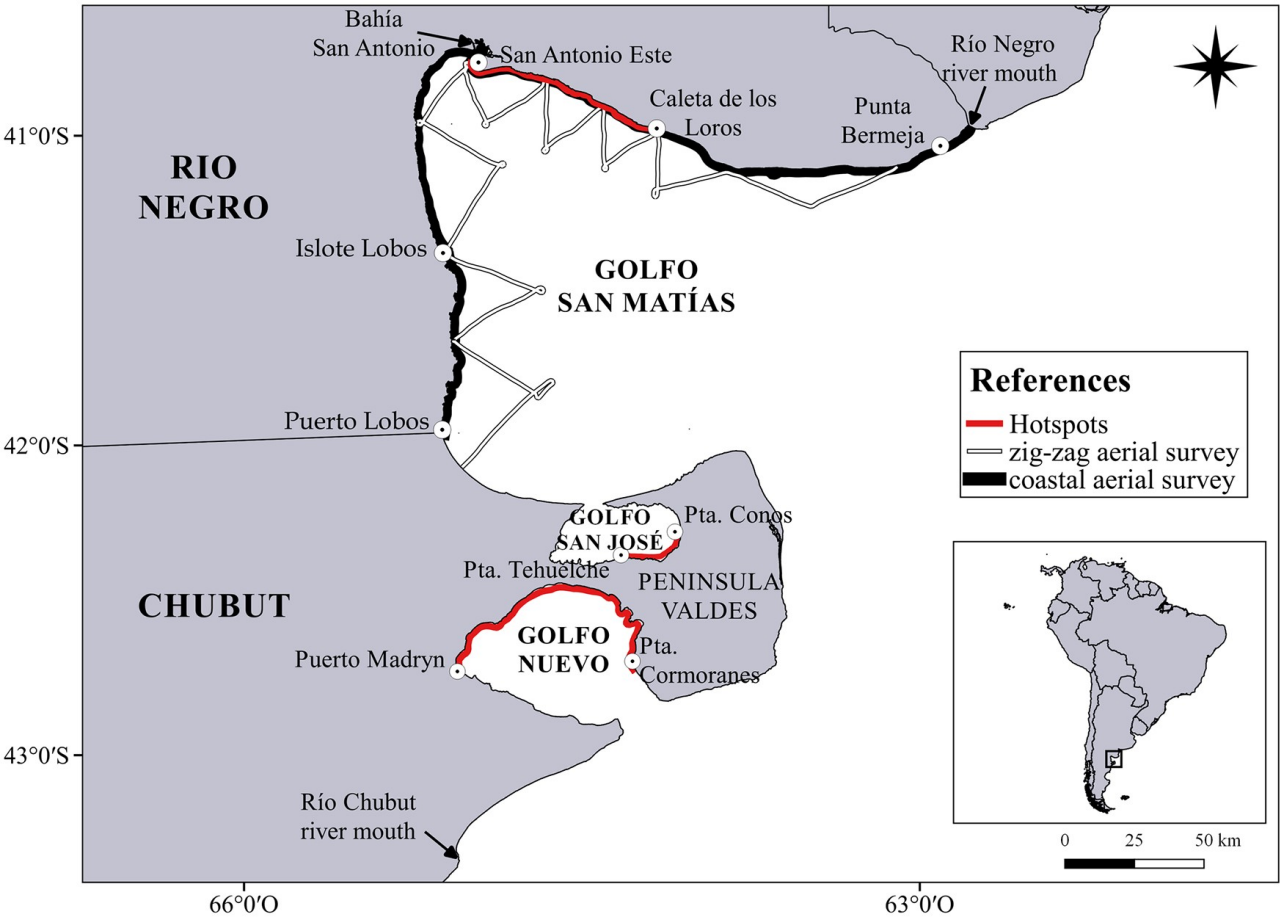
Study area, Golfo San Matías and Península Valdés system (Golfo San José and Golfo Nuevo), Argentina. The black line and the white one indicate the transects carried out during the coastal and zig-zag aerial surveys, respectively in the Golfo San Matías. The red areas indicate the hotspots in the respective gulf.

The study area encompasses 345 km of GSM coast and adjacent waters from Puerto Lobos (42° 00´S/ 65° 04´W) to the mouth of the Río Negro river (41° 02´S / 62° 47´W) (Fig 1), in Río Negro province, Argentina. The study area is adjacent to the main SRW nursery ground in South America; Península Valdés [10, 13, 24].

### Historical evidence of the presence of SRW in GSM

In the attempt to collect historical records of the presence of SRW in GSM, an online desktop literature review was completed. In addition, historical anecdotes were compiled on the presence of whales in the GSM. The information collected involves the review of historical records, commercial exploitation records and ecological monitoring data.

### Aerial surveys

**Fig 2:**
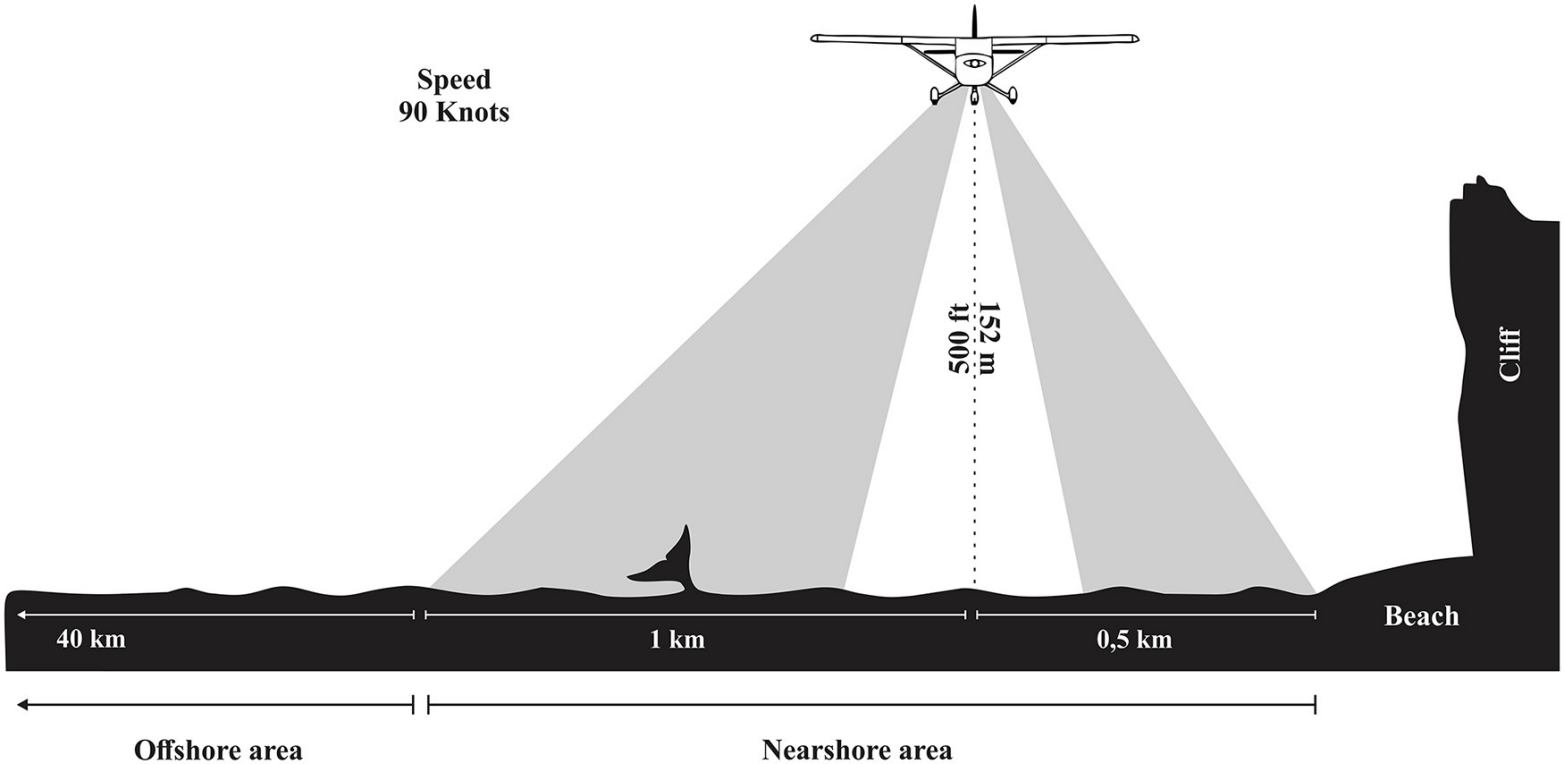
Methodology used to carry out the coastal aerial survey. In addition, the nearshore and offshore areas are shown in this figure.

Aerial surveys were carried out in GSM from 2007 to 2016 in order to evaluate the distribution and the relative abundance of SRW in the GSM coastal aerial. Permissions to perform these aerial surveys were issued by the Secretaría de Medio Ambiente y Desarrollo Sustentable of Río Negro province. There was no continuity of sampling throughout these years until 2013, when a minimum of one flight per season was completed annually. A total of thirteen coastal aerial surveys were conducted over 6 years. The timing of these surveys was planned for the period in which it is most likely to find right whales on the GSM coast, between August and October [17–19]. Surveys aim to record the position (lat, long) and group composition (the number of animals and the type of group) of SRW. Three group categories were identified: mother-calf pairs, one adult female with a calf; solitary individuals, adult or sub-adult males or females; breeding groups, two or more individuals socializing [20]. The flights were undertaken on a Cessna 182-B aircraft flying at a height of 500 ft (152 m) and 90 knots (170 km/h) when sighting conditions were good, without fog and with a sea state between 0 and 3 in the Beaufort scale. The width of the strip inlcuded 1.5 km: 0.5 km from the coast to the aircraft plus approximately 1 km from the plane to the open sea (Fig 2). Finally, the crew was composed of two observers, one on each side of the plane and a third person recording data.

### Validation of the coastal aerial survey methodology for the GSM

In order to validate the coastal aerial survey as a reliable indicator of the number of whales in the GSM, the relative abundance of whales in the “nearshore area” and in the “offshore area” were compared. The nearshore area was defined as the region between the coastline and a distance of 1.5 km from it and the offshore area as the region between 1.5 km and 40 km away from the coastline (Fig 2). For this survey, on September 18, 2015, a flight with a zig-zag pattern was carried out in GSM (Fig 1). The flight was performed with a Cessna 337 aircraft, using a similar protocol described above for coastal aerial surveys and flying at the same height and speed of the coastal aerial surveys. For this particular flight the locations of the whales were determined using distances to the transect measured with a clinometer. The distance to the coast was estimated for each whale with the QGIS software (version 2.18.4). Finally, to compare both areas, a relative sighting rate was calculated for whales seen in the nearshore area and in the offshore area and it was expressed as: “Sighting per Unit Effort” (SPUE): the number of whales sighted per kilometer of searching in each area.

### Evaluation of the distribution pattern

In order to evaluate the distribution pattern of SRW in GSM, the coastline of the GSM was divided into 69 segments of 5 km long by 1.5 km wide (width of the strip) with the QGIS software version 2.18.4 (www.qgis.org). The number of whales in each segment was recorded. The length of the segment was chosen following a previous methodology [13], which divided the coast into 5 km segments to evaluate the distribution of the SRW in Península Valdés.

With the purpose of comparing the SRW distribution throughout the years the aerial surveys made in the period of highest concentration of whales were used (late August—beginning of September) [17–19]. To avoid variations in the distribution that could be associated to the time in which each survey was undertaken, it was decided to compare surveys that coincided in time between years Thus, a total of four annual aerial surveys undertaken in 2007, 2014, 2015 and 2016.

To evaluate the presence of the different group types along the coast of the GSM, the number of whales belonging to the different group types in each segment was calculated.

### Population trend

For the analysis of population trend, the data collected in the thirteen coastal aerial surveys were used to estimate the trend of the number of whales that come to the GSM. During the 2013 census it was not possible to fly the entire GSM coast therefore, in order to reduce the potential bias caused by the differential effort in different areas of the GSM, only the area that was monitored in all the censuses was used for this analysis. This area was between Islote Lobos and Caleta de los Loros (Fig 1) and it coincides with the area where the right whales tend to be more abundant in the GSM [25]. The population trend was effectively estimated for this area.

A generalized linear model was built and the number of whales recorded in each flight was selected as the response variable. The structure of the model was the same that was used in a previous research project [20] to estimate the growth rate of this species in Península Valdés. Therefore, the Year, the Julian day and the Julian day^2^ were chosen as explanatory variables. In addition, considering that the sea state changed among flights, the Beaufort was added as an explanatory variable. The Beaufort was recorded in each of the 5 km coast segments mentioned above, and the median was estimated as a proxy for the conditions in each flight. The objective of this model building was to test if there is an effect of the year on the number of whales recorded in the coastal aerial surveys; hence we only considered the subset of models that included the explanatory variable Year plus the null model.

The models were constructed using a negative binomial error structure and were fitted using package *MASS* with the software R 3.3.1 [26]. The models were ranked according to the Akaike Information Criterion adjusted for small samples (AICc). Model comparisons were made with ΔAICc that indicate the magnitude of the difference in AICc values between each model and the model best supported by the data. Models with ΔAICc ≤ 2 were considered as candidate models for this data set [27]. Also we estimated the AICc weight value that is considered as the weight of evidence in favour of the model i, given the models considered [27]. Finally, a multi-model inference approach was performed using the functions *model*.*sel* and *model*.*avg* [27, 28].

### Analysis at regional scale

The proportion of the different group types was compared among northern Patagonian gulfs using a chi-square test. For this analysis, the area where the right whales tend to be more abundant in each gulf, hereafter called as “hotspot", was selected. In the case Península Valdés gulfs, the hotspots were chosen according to the available bibliography where two areas of greatest concentration have already been defined [13, 21, 22]: 1) the sector comprised between Puerto Madryn and Pta. Cormoranes, hereafter Golfo Nuevo hotspot, and 2) the sector comprised between Pta. Conos and Pta. Tehuelche, hereafter Golfo San José hotspot (Fig 1). In the case of the GSM, the hotspot was also chosen according to the available bibliography [17, 25], therefore, the hotspot was defined in the area between San Antonio Este and Caleta de los Loros (Fig 1), hereafter Golfo San Matías hotspot.

The database of GSM aerial surveys was compared with the database of the Península Valdés aerial surveys [20] that were performed using the same protocol described in this paper, and with the same aircraft and pilot. In order to carry out the comparison between the northern Patagonian gulfs selected flights were the ones that were close in time one to another (within few days). Thus, a considerable change in the number of whales in each area between these aerial surveys was not expected. A total of 11 flights were undertaken in the GSM and 11 were undertaken in Península Valdés.

In addition, the average number of whales per kilometre in each hotspot was evaluated. For this analysis a generalized linear model was developed with the software R 3.3.1 (*glm* in R package *lme4*) [26]. The response variable was the number of whales in each hotspot and the model was fitted with a negative binomial distribution. This distribution was used because the data showed over dispersion as it was expected. In addition, since the hotspots have different sizes, the kilometres surveyed were used in each hotspot as an offset variable. The explanatory variable was the hotspot and was treated as categorical. Contrast was made using the Tukey test.

## Results

### Historical evidence of the presence of SRW in GSM

The information collected involves the review of historical records, commercial exploitation records and ecological monitoring data. The historical records included the review of expeditions (navigators and explorers) who travelled along the coasts of the South West Atlantic Ocean and went through the coast of GSM during the whale season (July-December). Two expeditions that refer to the presence of SRW and whaling ships in GSM were found. The first expedition was made by the Captain Basilio Villarino between 1779 and 1781, who in his description of the west coast of GSM commented “*All along the coast between the port of San Jose and San Antonio*, *near countless whales are seen”* [29]. The second expedition was made by the Captain Benjamin Morrell, who in his passage through the port of Río Negro (Fig 1, mouth of Rio Negro river), in the north coast of GSM, on 9/21/1822 commented “*…our vessel being the first from the United States that ever entered to this river*. *(…) Rio Negro had been of very little note; but it is now much frequented*, *especially by whalers*, *who touch here for refreshments”* remarking the presence of whaling ships in the area. He even wrote directions to enter the Rio Negro river port, considering that it could be useful for the whaling ships that visited those coasts. Two days later, Benjamin Morrell arrived at the shores of Bahía San Antonio (Fig 1), and described it as follows: *“the bay itself is very convenient for whaling ships*, *particularly in the months of September*, *October*, *November*, *and December*, *when the whales come in to bring forth their young”* [30] again making reference to the whaling ships, and giving even more details of the seasonal presence of whales in the area. These are the first historical records that indicate the presence of SRW in GSM during the commercial whaling in the western Atlantic [1]. More recently, in the 20th century, there is another SRW record in GSM in middle July, 1969, when R. L. Brownell Jr. aboard the U.S. National Science Foundation’s R/V Hero recorded a SRW in the west of the entrance of Golfo San José, in GSM (Brownell Jr. pers. comm.).

In the case of the commercial exploitation records the main sources were the published articles that indicate the catch areas. According to our review no catch records were found in GSM, although as mentioned above, Benjamin Morrell referred to the presence of whaling ships in this area [30]. Nevertheless, there are several articles that describe the catches that occurred in these latitudes in offshore waters ("Bank Brazil/Islas Malvinas"), by the American, French, Spanish, British and Soviet whalers [1, 3, 31–36].

From the review of the scientific monitoring carried out in GSM, we found evidence of SRW presence in said area during the end of 20th century. The first record corresponds to studies (coast and boat based) undertaken between 1987–1992 [37]. In these studies the presence of SRW in the northwest of GSM between May and November, with peaks during July 1990 and August 1991, was reported. The highest number of whales counted in that period reached 59 individuals throughout an entire month (August 1991). Also, during the development of the on-board observer programme performed between 1994 and 1996, a solitary individual was recorded occasionally on August 21^st^, 1995, in the northwest of GSM (Gonzalez, unpublished data). The third record corresponds to Intrieri 1997 [38] who made coastal observations between 1993 and 1996 and reported an accumulated number of 179 SRW (31 mother-calf pairs and 148 solitary individuals—many of them could be the same individuals) in the north coast of GSM (surrounding Punta Bermeja).

### Validation of the coastal aerial survey methodology for GSM

**Fig 3:**
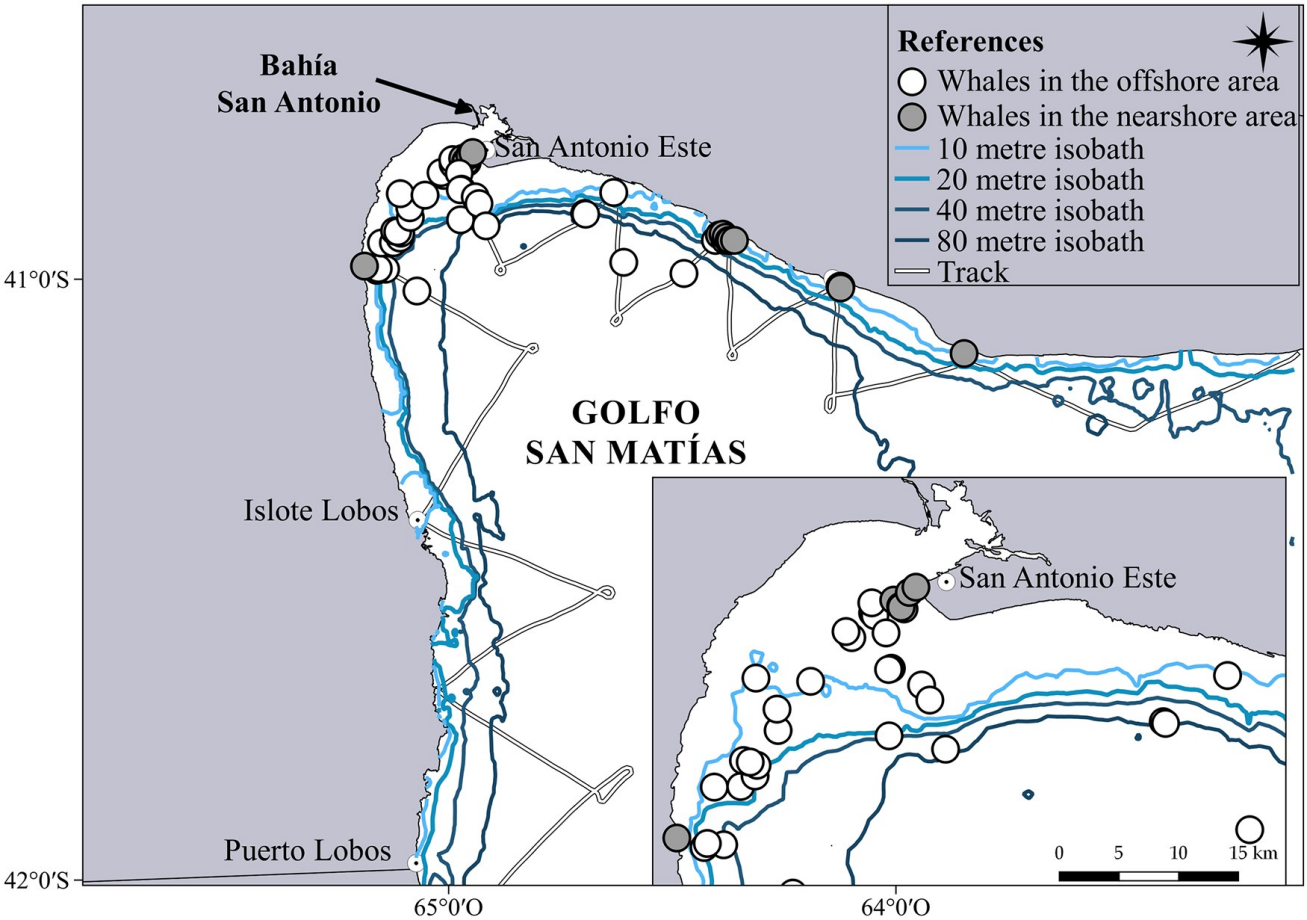
Location of the whales recorded in the zig-zag aerial survey, with a detail of the area around Bahía San Antonio. Each circle can indicate more than one right whale.

During the zig-zag aerial survey undertaken in September 2015 a total number of 81 whales were recorded, most of them on the northwest coast of GSM (Fig 3). The relative abundance of whales was higher in the nearshore area, with a SPUE more than eight times higher in the nearshore area than in the offshore area (Table 1). Even though the number of whales registered in the offshore area was greater, the 76.78% of them were located near Bahía San Antonio while in the rest of GSM most of the whales were found in the nearshore area (Fig 3).

**Table 1:** Detail of the zig-zag aerial survey and estimation of the Sighting per Unit Effort index (SPUE) for the nearshore and offshore areas.

	Nearshore area	Offshore area	Total
**Number of whales**	25	56	81
**Sampling effort (km)**	28.32	555.72	584.04
**SPUE**	0.88	0.10	0.14

### Aerial surveys

Whales were recorded on every flight undertaken between August and October since 2007 (Table 2). The highest number of whales was recorded in late August and early September, with a maximum of 160 individuals recorded in a single census in early September of 2015.

**Table 2:** Details of the aerial surveys, and the maximum number of whales registered on each flight. See Fig 1 for references.

Year	Dates	Maximun Num. of whales	Type of survey	Extent of survey
**2007**	23/8	56	Coastal	Puerto Lobos—mouth of the Río Negro river
**2007**	3/10	31	Coastal	Puerto Lobos—mouth of the Río Negro river
**2008**	5/10	51	Coastal	Puerto Lobos—mouth of the Río Negro river
**2013**	9/8	38	Coastal	Islote Lobos—Caleta de los Loros
**2013**	24/9	76	Coastal	Islote Lobos—Caleta de los Loros
**2014**	21/8	53	Coastal	Puerto Lobos—mouth of the Río Negro river
**2014**	11/10	23	Coastal	Puerto Lobos—mouth of the Río Negro river
**2014**	12/11	0	Coastal	Puerto Lobos—mouth of the Río Negro river
**2015**	2/9	160	Coastal	Puerto Lobos—mouth of the Río Negro river
**2015**	18/9	81	Zig-zag	Golfo San Matías
**2015**	9/10	9	Coastal	Puerto Lobos—mouth of the Río Negro river
**2016**	23/8	141	Coastal	Puerto Lobos—mouth of the Río Negro river
**2016**	23/9	112	Coastal	Puerto Lobos—mouth of the Río Negro river
**2016**	5/10	16	Coastal	Puerto Lobos—mouth of the Río Negro river

**Fig 4:**
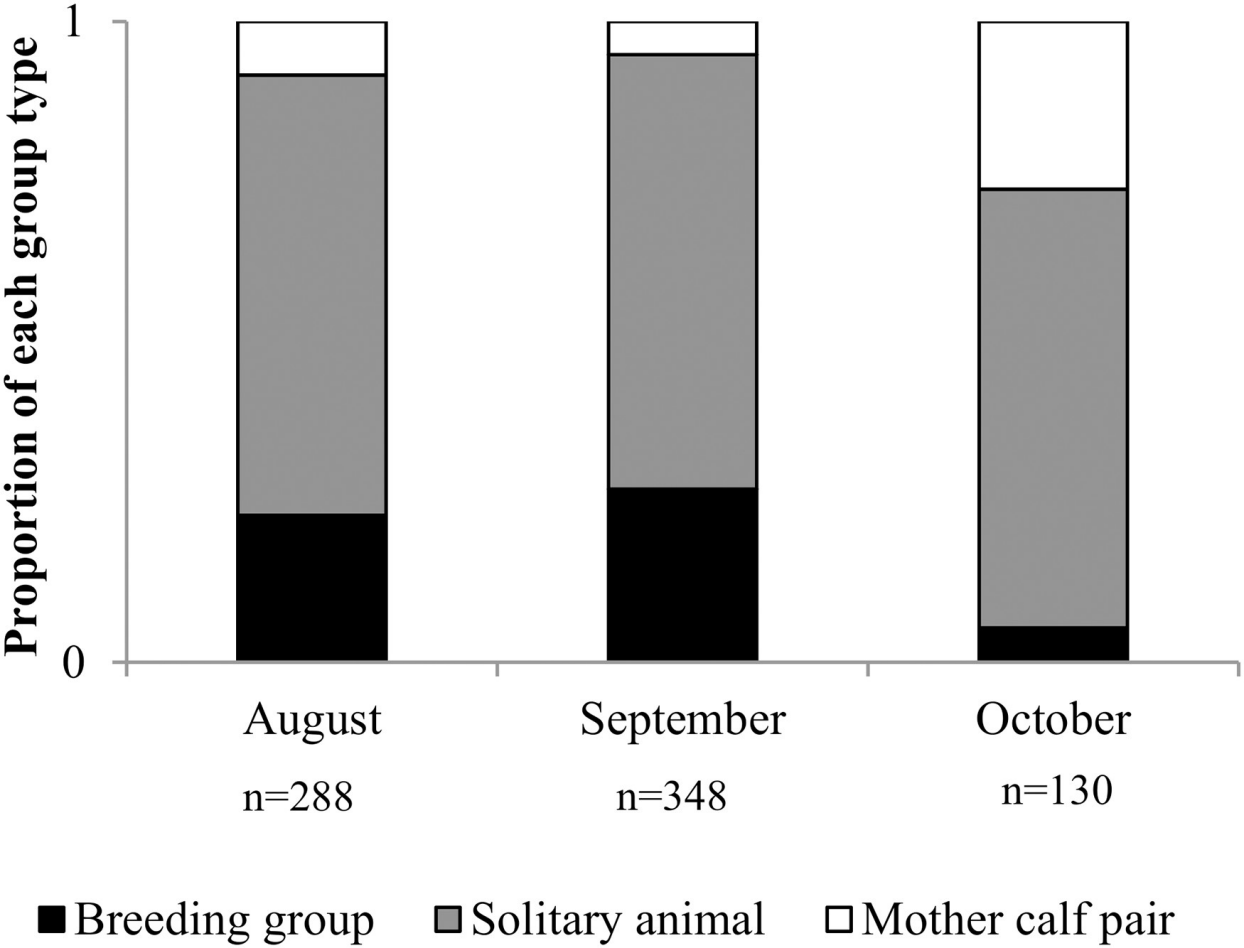
Changes in the proportion of whales in each group type observed throughout the season in GSM.

Considering all the surveyed years, a pattern of change in the frequency of group types throughout the season emerged (n = 766; X^2^ test = 63.79, gl = 4, *p*<0.0001) (Fig 4). The solitary individuals were always the predominant group type, but there was an increase in the frequency of: breeding groups, and; mother-calf pairs during September and October, respectively.

### Evaluation of the distribution pattern

**Fig 5:**
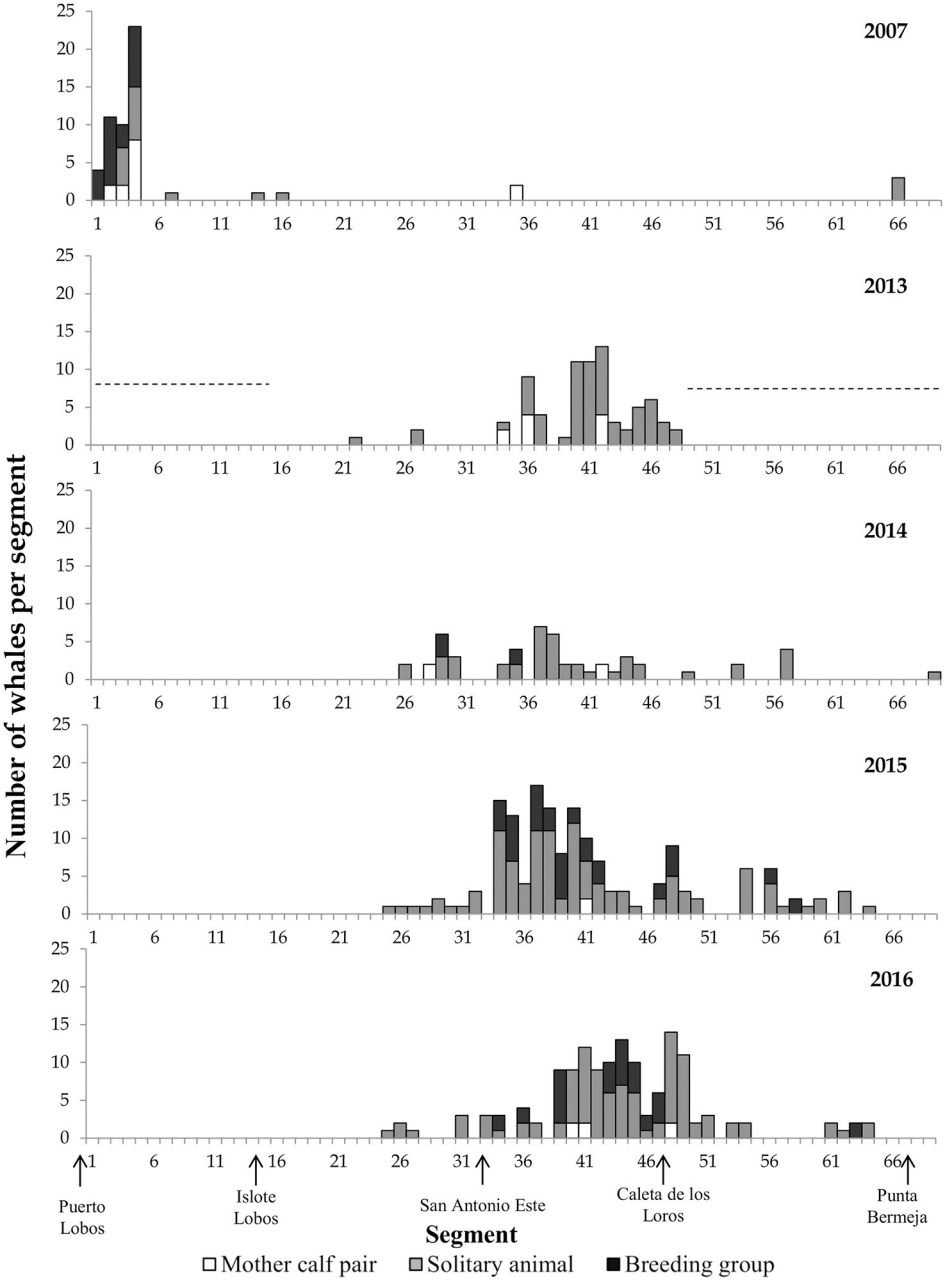
Group types and number of whales per segment recorded on the flight in which the highest number of whales was recorded each year. The dotted line in 2013 indicate the area that was not flown that year.

A change in the spatial distribution among years along the coast of GSM was recorded (Fig 5). In 2007, most whales were found around Puerto Lobos, near Península Valdés concentrated in a few segments (segments 1–4), and few whales were recorded on the north coast of GSM. During 2013–2016 the areas in which the whales concentrated changed and expanded. The SRW distribution was mainly confined to the northwest coast of GSM, particularly in the sector between San Antonio Este and Caleta de los Loros (Fig 5). From Caleta de los Loros towards to Punta Bermeja the presence of whales decreased.

The dominant group type in the coastal strip was the solitary individuals (Fig 5). Mother-calf pairs and breeding groups were mainly concentrated in the area around Puerto Lobos, near Península Valdés, and in the sector between San Antonio Este and Caleta de los Loros. Finally, the maximum density of whales was registered in 2007, with 3.06 whales/km^2^.

### Population trend

The best model supported by our data to account for the number of whales recorded in each aerial survey was the same model that was selected to estimate the population trend in Península Valdés [20], including the Year, the Julian day and the Julian day^2^. The growth rate estimated in each model was always positive with values that ranged between 8.25% and 17.12% and confidence intervals that ranged between -3.48% and 34.33%. The explanatory variable Year was significant only in the model that had the 4 covariates (Year + Julian day + Julian day^2^ + Beaufort). The inclusion of Beaufort in this model explained part of the variability that was associated to the different environmental conditions in which the flights were made. As a consequence of this inclusion, the effect of year became evident with and significant estimation of the growth rate of 13.36% (95% IC = 2.60%, 24.64%).

Although the first model and the second model had a ΔAICc > 2 no single model reached a threshold of AICc weight > 0.9 [27]. Therefore, to consider the variation among models a multi-model approach was performed. For this approach all the models that included the year and with a ΔAICc < 10 [27] were considered as candidate models. Coefficients revealed again a positive effect of Year, with an estimated growth rate of 10.02% (95%CI = -6.47%, 26.51%).

### Analysis at a regional scale

**Fig 6:**
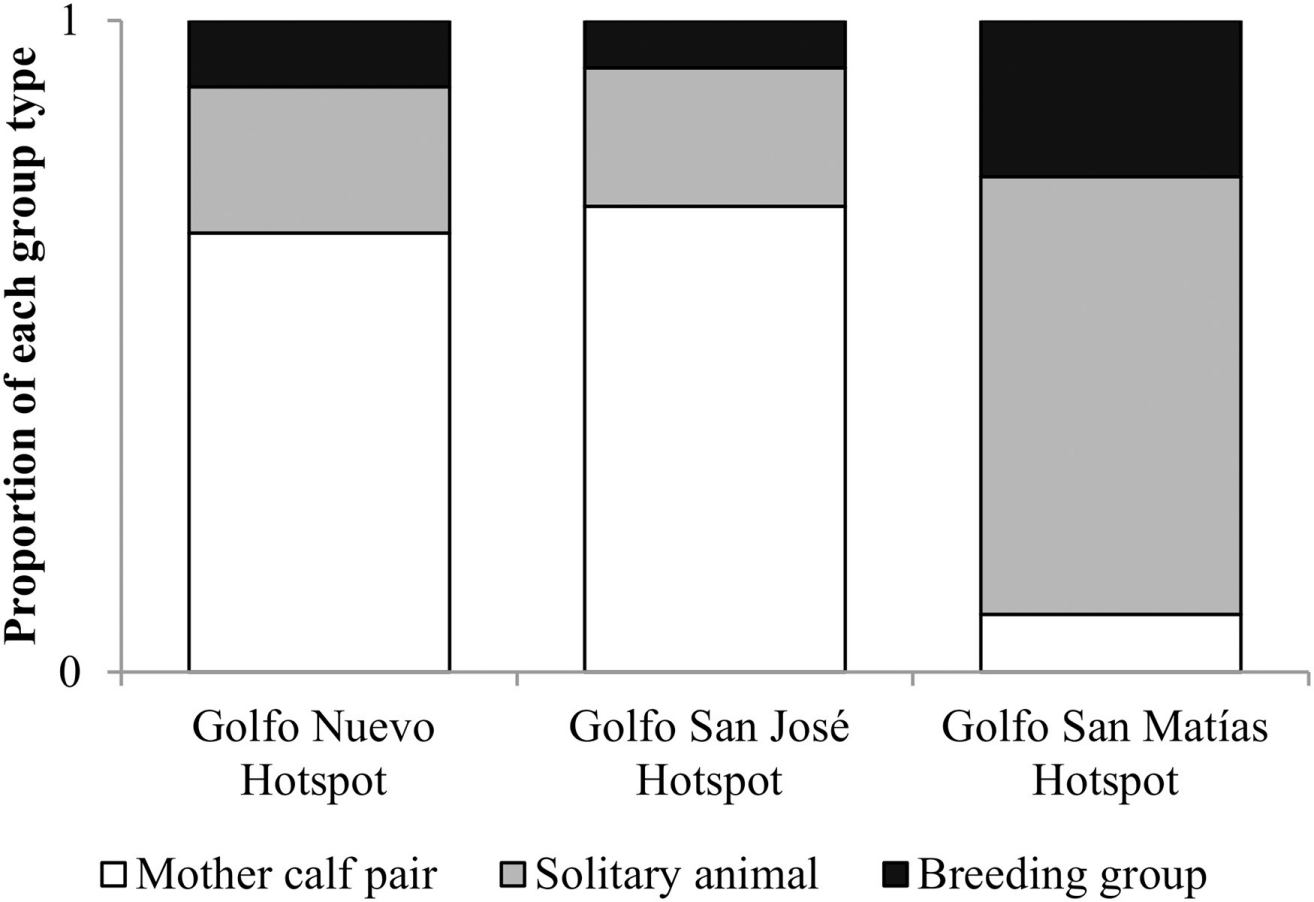
Proportion of whales in each group type observed in each hotspot.

There were significant differences in the proportion of group types observed in the different hotspots (n = 5926; X^2^ test = 745.36, gl = 4, *p* < 0.0001), with a dominance of mother-calf pairs in the Península Valdés hotspots (Golfo Nuevo hotspot and Golfo San José hotspot), and a dominance of solitary individuals in the GSM hotspot (Fig 6).

The generalized linear model developed for the analysis of the number of whales per kilometre surveyed in the different hotspots explained the 44.09% of the variation observed. Based on this model, no significant differences were observed in the relative abundance of whales estimated for the Golfo Nuevo hotspot and Golfo San José hotspot (Table 3), with an average of 3.09 and 3.70 whales/km, respectively. For the Golfo San Matías hotspot, the model estimates a significant lower number of whales per kilometre surveyed compared with the hotspots of Península Valdés with an average of 0.58 whales/km (Table 4).

**Table 3:** Parameter estimated, in the predictor scale, for the number of whales per kilometre surveyed in the different hotspots by the generalized linear model.

	Number of whales/km			
	Parameter Estimate	Standard Error	95% confident interval	*p*
Intercept (Golfo Nuevo Hotspot)	1.13	0.23	0.70, 1.62	< .001
Golfo San José Hotspot	0.18	0.33	-0.48, 0.83	0.594
Golfo San Matías Hotspot	-1.67	0.33	-2.33, -1.01	< .001

**Table 4:** Parameter estimated, in the predictor scale, for the Tukey contrast for the generalized linear model.

	Number of whales/km		
	Parameter Estimate	95% confident interval	*p*
Golfo San José Hotspot—Golfo Nuevo Hotspot	0.18	-0.60, 0.95	0.856
Golfo San Matías Hotspot—Golfo Nuevo Hotspot	-1.67	-2.45, -0.88	< .001
Golfo San Matías Hotspot—Golfo San José Hotspot	-1.85	-2.63, -1.06	< .001

## Discussion

This is the first study that presents quantitative evidence that the SRW is recolonising areas in north Patagonia. In particular, it explores the population trend of the SRW in GSM for the first time, and provides insight into the utilization of the under-surveyed northwest coast of GSM by right whales. To help focus this section we will: (1) discuss the distribution of whales in the nearshore and offshore areas; (2) summarize our observations in relations to seasonality, group composition and coastal distribution; (3) compare the population trend estimated for SRW in GSM to the surrounding areas; and (4) discussed the potential connection between the GSM and other wintering grounds.

### Presence of right whales in the nearshore and offshore areas

Our findings in relation to the SPUE analysis highlights the preference of SRW for areas near the coastline, supporting our assumption and it coincides with the previous knowledge in Península Valdés area when densities were low [10]. In GSM, most whales were found in the nearshore area, with the exception of the area adjacent to Bahía San Antonio. These results could be associated with the preference of the SRW for areas with depths less than 15 metres and they particularly select water depths of 5 metres [10]. Given that the surrounding area of Bahía San Antonio is characterized by shallow waters further from the coast due to sand beds [39, 40], the whales can find these depths in regions further away from the coast. Therefore, in this area the number of whales recorded by a coastal aerial survey could be underestimated. This should be considered when designing the next surveys, including some additional effort in this particular area. In the rest of GSM these depths are only found near the coast where most of the whales were recorded. A better estimate of the distribution and density of right whales in the area could have been done if a distance sampling analysis and density surface modelling were carried out. However, in this study the data to perform these analyses it was not available for the coastal aerial surveys. Therefore, the number of whales recorded in each coastal aerial survey should be considered indicative of relative, rather than absolute abundance.

### Seasonality, group composition and coastal distribution of SRW in GSM

The composition of groups observed in GSM was different from those observed in the Península Valdés system (Golfo San José and Golfo Nuevo), with a dominance of solitary individuals throughout the season and along all GSM coast consistent with previous studies for the area [21, 41]. The presence of mother-calf pairs and breeding groups showed a similar trend to those in Península Valdés [21], with breeding groups mainly present early in the season and mother-calf pairs growing towards its end.

The low number of mother-calf pairs observed in GSM could indicate that, at least at present, this is not a core area for rearing calves. However, considering the reports of Benjamin Morrell at the beginning of the 19th century [30], GSM and particularly the Bahía San Antonio area could have been an important breeding area at that time. In the same sense, Svendsen 2013 [25] using habitat models found that some places in the northwest area of GSM would constitute suitable habitats for mother-calf pairs of right whales. In recent years, there have been records of mothers with very young calves, sometimes with fetal folds. This indicates that these calves were born in GSM, particularly in the area around Bahía San Antonio (M.A. pers. obs.). Also, the presence of breeding groups in GSM suggests that this might be an important habitat for social and reproduction activities.

In GSM, SRW distribution was concentrated on certain areas and changed over the years expanding into new areas. These changes could be a result of an exploratory process associated with the population increase of the species [20] and with a selection of the north coast of GSM over other suitable areas [25]. Changes in the distribution over years have also been observed in Península Valdés [13, 22] and Brazil [42]. These changes might be related to factors such as individual preference, social cohesion or habitat disturbance [13].

The number of whales per segment in the areas of highest concentration of GSM reached values similar to the mean number of whales per segment found in Península Valdés between 1991 and 1997 [13]. However, the maximum density of whales observed during the peak of the season in GSM (3.06 whales/km^2^) is much lower than the maximum density recorded (15.87 whales/km^2^) for Península Valdés in recent years [22]. So it is expected that the density of whales will increase in the coming years in these sectors with *a priori* similar environmental conditions to those observed in Península Valdés.

### Population trend estimated for SRW in GSM

Our results support a positive population trend for the SRW in the northwest of GSM. However, considering the variability observed in the data, the estimate growth rate of 10.02% can only be regarded as preliminary. The large confidence intervals estimated in each model reflect this variability and highlight the importance of the long-term studies. The results presented in this study must be considered as the starting point of a long-term data series which will allow us to improve the estimation of the population growth rate in future years. The SRW population growth rate was estimated to be 7.5% [1] for the Southern Hemisphere, and between 7 and 8% in Península Valdés until 2007 [20, 21, 24]. After 2007 the rate of population increase in Península Valdés decreased, with the latest estimation being 0.54% [20]. Taking into account this information, our results may be considered as an indication of population growth in GSM, with an increase rate between 8% and 13%. Considering the low proportion of mother calf pairs recorded in GSM, it is unlikely that this growth rate is the result of the productivity of whales seen in GSM. In addition, growth rates of this magnitude have been observed in other regions such as southern Brazil (increase rate 14%) and the west coast of South Africa (increase rate of 13%), where it was suggested that these growth rates were not only the result of overall population growth, but also reflect immigration and seasonal movement between different wintering grounds [11, 14].

### Connection between GSM and other wintering grounds

Our results suggest the immigration from other wintering grounds, but where do these whales come from? Movements at a smaller scale were reported for southern right whale in other wintering ground as Australia [43] and South Africa [14, 44, 45], and New Zealand [8, 46]. In addition, the expansion from the focal dense area to new areas has already been reported in other marine mammals of Patagonia such as South American sea lions (*Otaria flavescens*) [47]. Moreover, this expansion was associated with a density-dependent process. Therefore, a possible explanation for the increase in GSM is immigration of whales from the main aggregation area in Argentina (Península Valdés) associated with the proposed expansion of the Península Valdés population towards other areas due to a density-dependent process [20, 22]. This hypothesis is supported by recent studies that use satellite transmitters implanted on SRW specimens in Península Valdés and GSM [48, 49]. Said studies have shown that this species makes trips between the three northern Patagonian gulfs (GSM, Golfo San José and Golfo Nuevo) in the same season. However, long-range movement have also been reported for this species [7, 45, 50, 51], therefore the contribution of whales from other wintering ground, as Santa Catarina (2000 km north of the GSM) cannot be ruled out. A comparison of catalogues from these three wintering grounds and the continuation of satellite telemetry studies to understand the movement patterns of these whales would help to know the degree of overlap between these areas.

The relationship between GSM and Península Valdés right whales is not well understood but there is recent substantial evidence for connectivity between these two areas, since recent studies have reported that right whales make trips between these areas in the same season [48, 49]. In addition, recent studies propose that Península Valdés is going through a density-dependent process and right whales are moving to other peripheral areas [20, 22]. The dominance of solitary individuals in GSM and mother calf pairs in Península Valdés could be associated with this process, where the only fraction of the population that is still growing in Península Valdés are the mothers with calves [20] that have been concentrated in the nearshore area displacing the other whales (solitary animals and breeding groups) to peripheral areas [22]. The mothers with calves prefer shallow areas close to the shore and with presence of other cow-calf pairs while the habitat choice is less important for the unaccompanied whales (solitary animals and breeding groups) [52, 53]. Therefore, it has been proposed that when a threshold density is reached in the nearshore area, solitary whales search new areas of lower density [22]. Another factor that can be considered is the differential dispersion of sexes associated with avoidance of inbreeding and increased access to mates [54]. It is argued that there is a direction of the sex bias in mammals’ dispersal as a consequence of the type of mating system. In the case of polygynous and promiscuous species of mammals, such as the SRW, juvenile males are the predominant dispersers [54–57]. In the case of mother-calf pairs, the mother has an additional energetic cost as she faces a great energy demand due to gestation or lactation [58]. Therefore, the familiarity with a given area is beneficial for them while whales without calves can move without this additional cost. The results of this study support the hypothesis mentioned above. However, the sex of the solitary individuals observed in GSM should be studied in future work to be able to test this hypothesis.

The expansion of the distribution range of SRW observed in the northern Patagonian gulfs was also reported in other regions as Brazil [11, 9], South Africa [14, 23] and New Zealand [7, 8]. However, the factors that influence dispersal are not necessarily the same. Whales expanded their range and showed evidence of recolonisation of ancient areas around the main nursery area. As it has been observed in GSM, these news areas are dominated by unaccompanied whales and seem to be important for feeding and socializing but not as a nursery area [11, 14].

This study presents evidence of an important presence of SRW in GSM more than two hundreds years ago, before and during commercial exploitation. The degree of reduction suffered by the stock in this area, as well as its historical social structure and distribution are unknown. Several indicators found in this study such as the presence of whales in GSM before and after commercial exploitation, the change in distribution and the positive trend in the population growth rate support the hypothesis of recolonisation.

The results of this study contribute to the knowledge of the presence and population status of the SRW in north Patagonia and the starting point of a long-term data series. This information will enable us to improve the estimate rates of increase for this area, and is important for developing management measures and decision-making related to the conservation of the species.

## Supporting information

S1 Table Position of the whales recorded during the zig-zag aerial survey. Latitude, longitude, number of individuals and angle measured with the clinometer.
(XLSX)

S2 Table Change in group composition throughout the season. Type and size (number of individuals) of group recorded during the coastal aerial surveys in Golfo San Matías in the different months. Group: Mother calf pair, Solitary animal, Breeding group; Month: August, September, October.
(XLSX)

S3 Table Number of right whales recorded on each coastal aerial survey between Islote Lobos and Caleta de los Loros. Data set used to estimate the population trend in Golfo San Matías, including the year and day (Julian day) in which the flight was made, the number of individuals recorded and the sea state (Beaufort scale).
(XLSX)

S4 Table Change in the distribution of right whales on the coast of Golfo San Matías. Group types and number of whales recorded per segment on the flight in which the highest number of whales was recorded each year. Group: Mother calf pair, Solitary animal, Breeding group; Segment: 1–69; Year: 2007, 2013, 2014, 2015, 2016.
(XLSX)

S5 Table Group composition in the different hotspots. Group types and total number of whales recorded per segment in each hotspot. The data were extracted from 11 flights performed in Golfo San Matías and 11 flights performed in Península Valdés. Hotspots: Golfo Nuevo, Golfo San José, Golfo San Matías; Group: Mother calf pair, Solitary animal, Breeding group.
(XLSX)

S6 Table Relative abundance of whales in each hotspot. Total number of whales recorded and kilometres survey in each hotspot in the 11 flights performed in Golfo San Matías that were compared with the 11 flights performed in Península Valdés. Hotspots: Golfo Nuevo, Golfo San José, Golfo San Matías.
(XLSX)
